# Selection Is a Significant Driver of Gene Gain and Loss in the Pangenome of the Bacterial Genus *Sulfurovum* in Geographically Distinct Deep-Sea Hydrothermal Vents

**DOI:** 10.1128/mSystems.00673-19

**Published:** 2020-04-14

**Authors:** Alief Moulana, Rika E. Anderson, Caroline S. Fortunato, Julie A. Huber

**Affiliations:** a Biology Department, Carleton College, Northfield, Minnesota, USA; b Department of Organismic and Evolutionary Biology, Harvard University, Cambridge, Massachusetts, USA; c Department of Biology, Wilkes University, Wilkes-Barre, Pennsylvania, USA; d Marine Chemistry & Geochemistry, Woods Hole Oceanographic Institution, Woods Hole, Massachusetts, USA; University of Georgia

**Keywords:** hydrothermal vents, metagenomics, pangenome

## Abstract

Microbial genomes have highly variable gene content, and the evolutionary history of microbial populations is shaped by gene gain and loss mediated by horizontal gene transfer and selection. To evaluate the influence of selection on gene content variation in hydrothermal vent microbial populations, we examined 22 metagenome-assembled genomes (MAGs) (70 to 97% complete) from the ubiquitous vent Epsilonbacteraeota genus *Sulfurovum* that were recovered from two deep-sea hydrothermal vent regions, Axial Seamount in the northeastern Pacific Ocean (13 MAGs) and the Mid-Cayman Rise in the Caribbean Sea (9 MAGs). Genes involved in housekeeping functions were highly conserved across *Sulfurovum* lineages. However, genes involved in environment-specific functions, and in particular phosphate regulation, were found mostly in *Sulfurovum* genomes from the Mid-Cayman Rise in the low-phosphate Atlantic Ocean environment, suggesting that nutrient limitation is an important selective pressure for these bacteria. Furthermore, genes that were rare within the pangenome were more likely to undergo positive selection than genes that were highly conserved in the pangenome, and they also appeared to have experienced gene-specific sweeps. Our results suggest that selection is a significant driver of gene gain and loss for dominant microbial lineages in hydrothermal vents and highlight the importance of factors like nutrient limitation in driving microbial adaptation and evolution.

**IMPORTANCE** Microbes can alter their gene content through the gain and loss of genes. However, there is some debate as to whether natural selection or neutral processes play a stronger role in molding the gene content of microbial genomes. In this study, we examined variation in gene content for the Epsilonbacteraeota genus *Sulfurovum* from deep-sea hydrothermal vents, which are dynamic habitats known for extensive horizontal gene transfer within microbial populations. Our results show that natural selection is a strong driver of *Sulfurovum* gene content and that nutrient limitation in particular has shaped the *Sulfurovum* genome, leading to differences in gene content between ocean basins. Our results also suggest that recently acquired genes undergo stronger selection than genes that were acquired in the more distant past. Overall, our results highlight the importance of natural selection in driving the evolution of microbial populations in these dynamic habitats.

## Abstract (precis)

Microbes can alter their gene content through the gain and loss of genes. However, there is some debate as to whether natural selection or neutral processes play a stronger role in molding the gene content of microbial genomes. In this study, we examined variation in gene content for the Epsilonbacteraeota genus *Sulfurovum* from deep-sea hydrothermal vents, which are dynamic habitats known for extensive horizontal gene transfer within microbial populations. Our results show that natural selection is a strong driver of *Sulfurovum* gene content and that nutrient limitation in particular has shaped the *Sulfurovum* genome, leading to differences in gene content between ocean basins. Our results also suggest that recently acquired genes undergo stronger selection than genes that were acquired in the more distant past. Overall, our results highlight the importance of natural selection in driving the evolution of microbial populations in these dynamic habitats.

## INTRODUCTION

Microbial populations can adapt to their environment through the acquisition of genes via horizontal gene transfer (HGT), enabling the introduction of novel functions (1, 2). Because HGT facilitates genetic exchange across distinct phylogenetic clades and even domains (3, 4), its common occurrence allows microorganisms to develop high variation in gene content within individual species or genera (5, 6). Although some gene content variation arises during duplication and gene loss events, HGT is hypothesized to be the primary mechanism of novel gene acquisition (1). This acquisition allows for some genes, called accessory genes, to be present in only a few strains, but not all strains, of a given species (7). In contrast, genes that belong to the core genome are mostly inherited and conserved across almost all strains within the species. The pangenome of a given microbial species or genus includes the sum of all core and accessory genes identified within that genus or species (7, 8).

The evolutionary processes that drive the accumulation of accessory genes in a microbial species in any system are still debated. Daubin and Ochman (10) show that compared to the core genome, the accessory genome of Escherichia coli has a significantly higher ratio of nonsynonymous (altering the amino acid sequence) to synonymous (not altering the amino acid sequence) nucleotide substitutions in an alignment of sequences. This difference suggests that the accessory genome must undergo much weaker, or neutral, selection compared to the highly conserved core genome. Consequently, it was suggested that neutral processes, such as genetic drift rather than natural selection, drive the accumulation of accessory genes. More recently, Andreani et al. (11) argued for the neutral evolution of pangenomes by showing a significant positive correlation between genome fluidity, a measure of dissimilarity in gene content within species, and effective population size. They argued that because larger effective populations had more pangenome diversity, this was consistent with the expectation that larger populations should have more genetic diversity due to neutral evolution (12). In contrast, other evidence suggests that selection, rather than neutral processes, is a strong factor in driving pangenome evolution. Microbes with similar ecology tend to share the same genes, even after controlling for phylogeny and geography, suggesting that novel genes acquired through HGT are maintained by selection pressures from the local ecosystem. For example, an analysis of 657 sequenced prokaryote genomes revealed that highly connected donors and recipients in transfers are of the same general habitat (13). Similarly, in the human microbiome, a network of 10,770 recently transferred genes was also shaped by ecological niche, and in particular, body site location (14). Further, acquired accessory genes have enabled bacteria to survive in extreme environments (15–17), suggesting that the acquired genes allow their hosts to exploit new habitats. Thus, it remains an open question whether the accumulation and retention of accessory genes are generally driven by natural selection or neutral processes.

In cases where selection is the primary driver for the accumulation of accessory genes, one would expect selection signatures to be present in these genes. A selective sweep, for instance, is known to occur in microbial populations when a genotype has a fitness advantage and sweeps through the whole population along with its associated genome (18). However, despite the support for this phenomenon in theoretical models (19, 20) and laboratory settings (21, 22), genome-wide selective sweeps have rarely been observed among environmental microbes (23, 24), and a high genetic diversity has been found in the core genome within species (25–27). Instead of sweeping at the genome-wide level, individual genes within a region could sweep independently of the rest of the genome, a phenomenon that has been observed, for instance, in the *gapA* and *pabB* loci of E. coli (28) and in single locus variation sites in the haloarchaeal genus *Halorubrum* (29, 30). Such patterns of gene-specific sweeps are hypothesized to emerge from high recombination rates, which can unlink a gene from the rest of the genome (31). Other hypotheses suggest that negative frequency-dependent selection constrains the adaptive-acquired genes so that they, and the recipient genome, do not sweep the entire population (24, 32). In this case, top-down control exerted by phages could selectively remove individuals that rise to high frequency because of greater fitness (33).

In order to identify the drivers of evolution in accessory genes in a microbial population in nature, we examined the evolutionary dynamics of genome variation in deep-sea hydrothermal vent habitats, which are powered by rich chemical redox gradients that support diverse microbial communities (34–37). In hydrothermal vent systems, HGT is extensive and facilitates the accumulation of accessory genes in microbial pangenomes in the ecosystem (38–41). A high diversity of other microorganisms in close proximity to one another may allow for interspecies transfers to occur, and the high abundance of viruses at vents provides another mechanism for such horizontal gene transfer to take place (42, 43). Moreover, organisms have to adapt to rapidly changing conditions in the gradient-driven environments of deep-sea hydrothermal vents (43–46), making it an ideal environment to study the evolutionary dynamics of genome variation.

Despite the prevalence of HGT and the unique challenges presented to microorganisms native to the vent habitat, very few studies have examined gene flow and biogeographic structuring of microbial populations in hydrothermal systems. Work on macrofauna in deep-sea vent sites has indicated that the degree of gene flow between sites varies widely depending on the species and location (47), but few studies have focused on the metapopulation structuring of microbial populations in this environment. Work based on multilocus sequence typing (MLST) to characterize specific microbial strains has demonstrated wide dispersal for vent microbes in general, and a correlation between genetic and geographic distance (48). Little is known about biogeographic structuring of other microbial populations at hydrothermal vents, and no studies have investigated the degree to which gene flow molds microbial pangenomes in this habitat. Therefore, such studies are crucial for understanding how evolution molds microbial lineages across space and time at these globally distributed, highly productive deep-sea environments (49).

We specifically studied the structure of the *Sulfurovum* pangenome using metagenome-assembled genomes (MAGs) collected from low-temperature diffuse fluids from two vent fields, one in the Caribbean Sea and the other in the Pacific Ocean. The *Sulfurovum* genus is a diverse group of mesophilic, sulfur-oxidizing Epsilonbacteraeota that is ubiquitous and abundant in deep-sea hydrothermal vent fluids across the world’s oceans (27, 50). Previous work has shown that sulfur-oxidizing bacteria in general demonstrate niche partitioning across fluid gradients in deep-sea hydrothermal vents and other sulfide-rich marine ecosystems, such as anoxic basins (36, 51–53). Work based on both 16S rRNA gene and metagenomic sequencing has revealed that the *Sulfurovum* clade in particular exhibits extensive genomic diversity, and it has been hypothesized that this is due to the steep geochemical gradients that are characteristic of the habitats where *Sulfurovum* has been found (27, 51, 54). Others have examined the nature of genomic variation in the *Sulfurovum* clade, revealing intraclade variation in the *sox* gene complex, oxygen- and nitrogen-cycling genes (51, 54). Although previous studies have demonstrated some genomic variation governing metabolic capabilities among Epsilonbacteraeota in general (55) and *Sulfurovum* in particular (51, 54), here we focused on biogeographic structuring and signatures of selection on the *Sulfurovum* pangenome (55). We investigated evolutionary dynamics on the *Sulfurovum* pangenome in two geographically distinct hydrothermal vent habitats to determine what, if any, selection pressures are present by assessing functional differences in genomes, computing selection strength, and searching for evidence of selective sweeps.

Our first study site is the Mid-Cayman Rise site in the Caribbean Sea, an ultraslow spreading ridge which harbors two geochemically distinct hydrothermal vent fields along the ∼110-km-long ridge: Von Damm (∼2,350 m), an ultramafic-hosted vent field, and Piccard (∼4,950 m), a deep, mafic-hosted vent field (56). Although these vent fields are substantially different in terms of depth and geological context, both vent fields host fluids rich in hydrogen and hydrogen-utilizing microbes (50, 56, 57). The second site is Axial Seamount, an active submarine volcano on the Juan de Fuca Ridge in the northeastern Pacific Ocean, which is home to multiple vent fields within the caldera. Hydrogen sulfide oxidation is the dominant chemical energy source for microbial metabolism at Axial Seamount, and the availability of hydrogen and nitrate influences the metabolism of these diverse microbial communities (34, 58–61). Here, using metagenomic techniques, we study pangenome evolution in two geographically and geochemically distinct hydrothermal vent systems and demonstrate the significance of selection in the maintenance of accessory genes in the pangenome.

## RESULTS

### Gene content variability among 22 *Sulfurovum* MAGs.

We collected a total of 159 MAGs from Axial Seamount and 77 MAGs from the Mid-Cayman Rise. The taxonomic identification of the MAGs recovered from the two vent fields were different (see Fig. S1A in the supplemental material), and the collection of MAGs recovered from Axial Seamount had a higher species richness than those from the Mid-Cayman Rise. While both vent fields were dominated by *Sulfurovum*, the taxonomic compositions then diverged. *Aquificales*, *Thiotrichales*, and *Sulfurovum* made up at least one-third of the microbial community recovered from the Mid-Cayman Rise samples. In contrast, despite the abundance of *Arcobacter* and *Sulfurovum* in the Axial samples, other taxa with much lower frequency were present at Axial Seamount. Due to the high abundance of *Sulfurovum* in both fields, we decided to use this taxon for our subsequent pangenomic analyses.

10.1128/mSystems.00673-19.1 FIG S1 Taxonomy of MAGs. (A) The taxonomic distribution of MAGs in the two vent fields. The stacked bars represent the relative distribution of each taxon (to the genus level) in each vent field. The taxa are arranged from the most at the top to the least abundant at the bottom in the Axial Seamount field. (B) Phylogenomic tree depicting phylogenetic relationships among *Sulfurovum* metagenome-assembled genomes (MAGs) and reference strains. MAGs recovered from Axial Seamount are shown in gray. MAGs recovered from the Mid-Cayman Rise are shown in orange. Reference strains are shown in blue. Alignments were generated by PhyloSift, and the tree was generated in RAxML. Download FIG S1, PDF file, 0.3 MB. Copyright © 2020 Moulana et al. 2020 Moulana et al. This content is distributed under the terms of the Creative Commons Attribution 4.0 International license.

**FIG 1:**
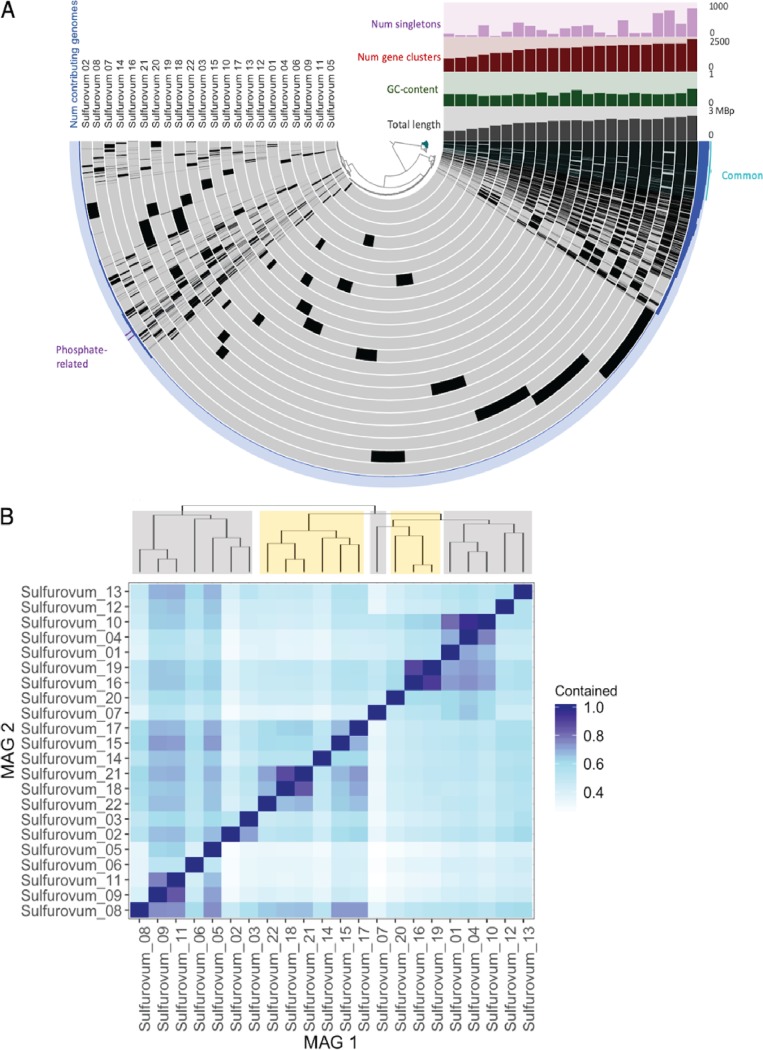
Pangenomic structure of *Sulfurovum* populations in the two vent fields. (A) Anvi’o image of each *Sulfurovum* metagenome-assembled genome (MAG) represented by a gray ring in the circle, ordered from MAGs with the most (outermost) to the least (innermost) gene clusters. The black boxes in each genome represent open reading frames (ORFs) recovered in that MAG. Boxes that align create a gene cluster for that ORF. The outermost ring in blue represents the number of MAGs containing the corresponding ORF. Other properties are shown in the upper right quadrant, where “num singletons” represents the number of genes found in only one MAG and “num gene clusters” represents the number of total gene clusters found in that MAG. The most common gene clusters (green) and phosphate-related gene clusters (blue) are highlighted. (B) Proportion of gene clusters present in one *Sulfurovum* MAG compared to every other *Sulfurovum* MAG. MAGs are hierarchically clustered by these proportions. Gray branches show Axial Seamount lineages, whereas golden branches show Mid-Cayman Rise lineages.

We examined 22 *Sulfurovum* MAGs (13 from Axial Seamount and 9 from the Mid-Cayman Rise) to create a pangenomic profile of *Sulfurovum* in the two vent fields (Fig. 1A). A phylogenomic tree of all *Sulfurovum* MAGs based on universal single-copy genes (Fig. S1B) indicates that MAGs from Axial Seamount and the Mid-Cayman Rise did not cluster entirely separately but that MAGs from the same location tended to cluster together within clades. We treated each MAG as a population of the same (or similar) *Sulfurovum* strains. The MAGs had similar GC content (mean, 36.44; maximum [max], 50.57; minimum [min], 29.79) and genome size (mean, 1.6 Mbp; max, 2.2 Mbp; min, 875 kbp) (see Table S2 in the supplemental material). The open reading frames (ORFs) in all *Sulfurovum* MAGs were clustered based on their amino acid sequence similarities (see Materials and Methods). For each gene cluster, we counted the number of genomes the gene cluster was found in and defined this count as gene frequency across MAGs.

In total, 10,263 gene clusters were identified, formed by 39,120 unique ORFs. On average, ∼4.6% of the genes in the genome were shared by all MAGs, and 83 gene clusters constituted the core genome (Fig. 1A). The addition of more genomes into the analysis would likely lead to a higher proportion of accessory genes because the *Sulfurovum* pangenome, as defined by these MAGs, is open (Fig. S2A). It is important to note that due to the incompleteness of the MAGs recovered (70 to 97%), the core genome is likely much larger, with core genes missed in our analysis. To examine this, we estimated the probability of an accessory gene being found in all MAGs as a core gene and determined that genes identified in 21 and 22 MAGs had, on average, a probability of 0.0604 and 0.2554, respectively, to be found in all *Sulfurovum* genomes (Fig. S2B). This probability declined for lower-frequency genes. Moreover, some genes found in all MAGs may in reality be missing in some of the MAGs due to redundancy, which ranged from <1 to 5% for the *Sulfurovum* MAGs. Thus, here we focus on the relative frequency at which a specific gene cluster is found across MAGs and do not adhere to a strict definition of what constitutes an accessory and core gene.

10.1128/mSystems.00673-19.2 FIG S2 Core genes in the *Sulfurovum* pangenome. (A) Number of total and core genes in *Sulfurovum* MAGs recovered. The total number of gene clusters and core genes found after observation of a certain number of MAGs is plotted against the number of MAGs that is observed. (B) Probability simulation of an accessory gene belonging to the core genome. The probability (log_10_ transformed) was plotted against the number of *Sulfurovum* MAGs the gene is found in. Download FIG S2, PDF file, 0.1 MB. Copyright © 2020 Moulana et al. 2020 Moulana et al. This content is distributed under the terms of the Creative Commons Attribution 4.0 International license.

Pairwise comparison among the MAGs showed a wide range of gene content similarity (from 0.25 to 0.96; Fig. 1B). Based on this gene content similarity, MAGs from the same region (Axial Seamount or Mid-Cayman Rise) tended to cluster together, but not exclusively (Fig. 1B). Further, the asymmetry of this similarity matrix highlights the different genome sizes and completeness of the MAGs. For instance, although 95% of genes present in Sulfurovum_10 were contained in Sulfurovum_04, only 76% of Sulfurovum_04 genes were in Sulfurovum_10 (Fig. 1B).

### Functional differences between high- and low-frequency genes in the *Sulfurovum* pangenome.

**FIG 2:**
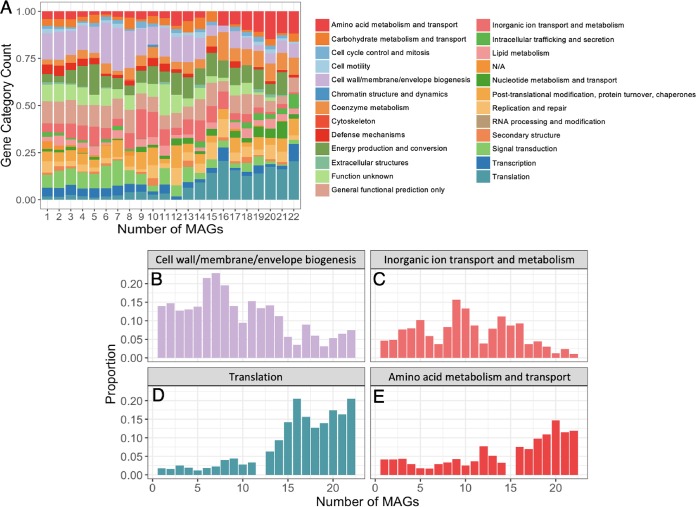
ORF annotations across the *Sulfurovum* metagenome-assembled genomes (MAGs) as a function of gene cluster frequency. (A) Each bar represents a cluster of orthologous groups (COG) category for the gene cluster of interest in the *Sulfurovum* MAGs. Each color corresponds to each COG category. Gene clusters with unknown annotations are excluded. N/A, no COG category assigned. (B through E) The proportion of ORFs that are in each of four COG categories is shown as a function of the gene cluster frequency (number of MAGs that contains the gene cluster). The *x* axis represents the number of MAGs sharing a specific gene cluster. The *y* axis represents the proportion of gene clusters that fall within a specific COG category out of all gene clusters shared among that number of MAGs. These four COG categories were included because they exhibited the strongest differences in abundance between high-frequency and low-frequency genes.

Gene annotation revealed functional differences between high- and low-frequency genes in the *Sulfurovum* pangenomes. To simplify, we put each gene annotation into its corresponding cluster of orthologous groups (COG) category. The distribution of these COG categories varies across gene cluster frequency in the *Sulfurovum* pangenome (Fig. 2A). For instance, translation (COG category J) and amino acid metabolism (COG category E) were significantly enriched in the highly conserved, higher-frequency genes compared to lower-frequency genes (Fig. 2D and E; Fig. S3; *P* < 0.0001). Other categories that were enriched in higher-frequency genes included coenzyme metabolism (COG category H) and nucleotide metabolism and transport (COG category F) (Fig. S3). The translation functions enriched in the higher-frequency genes included ribosomal proteins, 16S rRNA, and tRNA synthetases (see Data Set S1A in the supplemental material). Genes involved in other housekeeping functions, especially those related to transcription, electron transport, and general cell cycle, were also common functions for high-frequency genes in the *Sulfurovum* pangenome.

10.1128/mSystems.00673-19.3 FIG S3 *P* values of category distribution across frequency. Each square in the matrix represents the frequency that a COG category is represented more in genes of a specific frequency than expected based on nonparametric simulations (see Materials and Methods). Red squares represent values less than 0.0001 (light to dark: high to low) and blue values greater than 0.9999 (light to dark: low to high). For both of these colors, the *P* values are less than 0.0001. White squares represent *P* values between 0.999 and 0.0001. A dendrogram is displayed to represent the hierarchical clustering among COG categories based on these *P* values. Download FIG S3, PDF file, 0.2 MB. Copyright © 2020 Moulana et al. 2020 Moulana et al. This content is distributed under the terms of the Creative Commons Attribution 4.0 International license.

10.1128/mSystems.00673-19.10 DATA SET S1 COG function enrichment in low- and high-frequency genes. All genes found in at least 15 MAGs with J category (translation) function (A) and singleton genes (B) with M category (membrane/cell wall biosynthesis) are listed along with their corresponding functions. Download Data Set S1, XLSX file, 0.2 MB. Copyright © 2020 Moulana et al. 2020 Moulana et al. This content is distributed under the terms of the Creative Commons Attribution 4.0 International license.

In contrast, genes with functions relating to cell membrane/wall/envelope biogenesis (COG category M), signal transduction (COG category T), and inorganic ion transport and metabolism (COG category P) were significantly more common in lower-frequency genes relative to higher-frequency genes within the *Sulfurovum* pangenome (Fig. 2B and C; Fig. S3; *P* < 0.0001; *t* test). A large amount of singleton genes in the cell membrane/wall/envelope biogenesis category (COG category M), for instance, coded for glycosyltransferase involved in cell wall biosynthesis (Data Set S1B). Other common functions in genes found in <15 MAGs included transposases, ABC-type transport systems, and surface antigens. In general, outer membrane proteins made up a significant proportion of the lower-frequency genes. Further, the genes related to inorganic ion transport and metabolism (COG category P) were uniquely enriched among the medium-frequency genes (found in 9 to 10 MAGs) (Fig. 2C). Genes related to carbohydrate metabolism and transport (COG category G) shared a similar pattern to genes in the P category (inorganic ion transport and metabolism).

**FIG 3:**
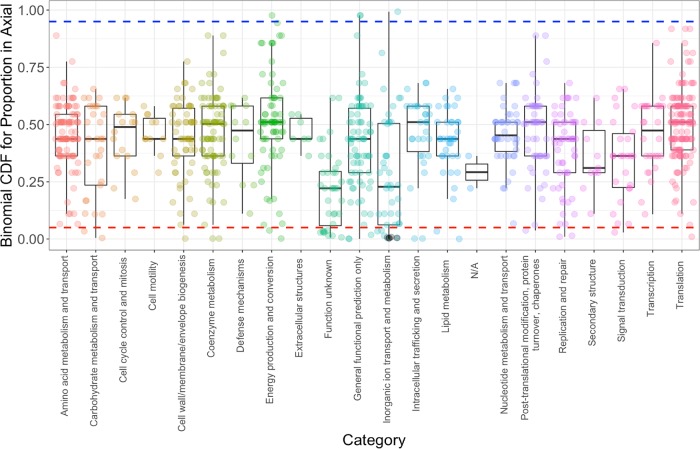
The cumulative distribution function (CDF) value for the proportion of genes present in Axial Seamount metagenome-assembled genomes (MAGs) relative to Mid-Cayman Rise MAGs across different clusters of orthologous groups (COG) categories. A higher value means that the open reading frame (ORF) is found in more Axial MAGs than expected, and vice versa. Some COG categories are not shown because they were not represented in enough MAGs (see Results). The blue dashed line represents a CDF value of 0.95, and a statistical significance cutoff for ORFs represented more in Axial MAGs (*P* < 0.05). The red dashed line represents the cutoff for ORFs represented more in Mid-Cayman Rise MAGs. Phosphate-related genes are shown in black under “Inorganic ion transport and metabolism.”

We next sought to determine whether specific genes were enriched in *Sulfurovum* genomes at either Axial Seamount or the Mid-Cayman Rise. For each gene cluster, we tallied all MAGs in which the cluster was found and then calculated the number of those MAGs that were recovered from Axial Seamount. Then, we normalized the proportions of gene clusters found in Axial Seamount by calculating their respective binomial cumulative distribution function (CDF) values with an expected value of 13/22 (see Materials and Methods; Fig. 3). The CDF is effectively the probability that the actual number of Axial Seamount MAGs that a gene cluster is found in is higher than would be expected randomly. To avoid bias driven by lower-frequency genes, only gene clusters that were found in at least 7 Mid-Cayman Rise MAGs or 12 Axial MAGS were included. This also ensured that the genes included in the analysis were conserved in at least one of the vent fields (i.e., these genes are at least medium-frequency genes). Although the proportions for most of the gene clusters were similar and concentrated around the expected value of 0.5, gene clusters related to inorganic ion transport and metabolism (COG category P) and unknown function (COG category S) had significantly higher representation at the Mid-Cayman Rise compared to Axial Seamount (*P* = 0.0005 and *P* < 0.0001, respectively; *t* test, Fig. 3). Of the 15 P category genes that were enriched at the Mid-Cayman Rise, four of them were related to phosphate uptake and regulation (Table 1). Three of these functions make up the components of ABC-type phosphate transport system. Moreover, arsenate reductase genes and some heavy metal transporters were more highly represented in *Sulfurovum* MAGs from the Mid-Cayman Rise compared to Axial Seamount. In contrast, only three gene clusters had significantly higher representation in the Axial MAGs (CDF > 0.95), with two of them being DNA-binding beta-propeller fold protein YncE (Fig. 3), which is known to be involved in iron metabolism (62).

**TABLE 1:** COG categories for inorganic transport and metabolism functions with the lowest representation in the Axial Seamount genomes^*a*^

Cluster ID	COG function(s)	Axial proportion
GC_00001517	Outer membrane receptor proteins, mostly Fe transport; outer membrane cobalamin receptor protein	0.00191752
GC_00000911	Truncated hemoglobin YjbI	0.00449269
GC_00001253	ABC-type phosphate transport system, periplasmic component	0.00449269
GC_00001177	ABC-type phosphate transport system, ATPase component	0.00449269
GC_00001293	ABC-type phosphate transport system, permease component	0.00449269
GC_00001150	Phosphate uptake regulator	0.00449269
GC_00001267	ABC-type phosphate transport system, permease component	0.00984906
GC_00001114	Copper chaperone CopZ	0.01435327
GC_00001019	Arsenate reductase and related proteins, glutaredoxin family	0.03377563
GC_00000935	Arsenate reductase and related proteins, glutaredoxin family	0.03736214
GC_00001144	Adenylyl- and sulfurtransferase ThiI participates in tRNA 4-thiouridine and thiamine biosynthesis; rhodanese-related sulfurtransferase	0.06183014
GC_00001031	Cu/Ag efflux pump CusA	0.06183014
GC_00000853	Exopolyphosphatase/pppGpp-phosphohydrolase	0.06711717
GC_00000940	Divalent metal cation (Fe/Co/Zn/Cd) transporter	0.06711717
GC_00000827	Copper oxidase (laccase) domain	0.10814304

a Fifteen functions from the COG P category with the least representation are presented along with each of their cluster identifiers (IDs) in the pangenome and proportion value.

### Signatures of selection in the pangenome.

**FIG 4:**
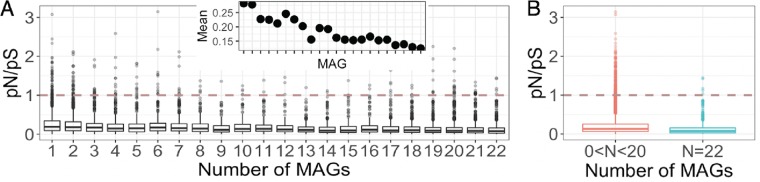
(A) The estimated *pN*/*pS* ratio of each identified open reading frame (ORF) within each gene cluster as a function of gene cluster frequency in the pangenome. The *x* axis represents the number of metagenome-assembled genomes (MAGs) sharing a specific gene cluster. The inset in panel A shows the trend of the mean of *pN*/*pS* ratios from lowest to highest frequency. The *x* axis is the same as in the larger figure. The dashed red line represents the value of *pN*/*pS* = 1. A similar plot is shown in panel B, where all ORFs with certain frequencies are grouped together (*N*), showing that ORFs within core gene clusters have lower *pN*/*pS* ratios.

In order to determine whether the *Sulfurovum* pangenome showed evidence of natural selection, we estimated the *pN*/*pS* ratios of each of the 39,120 identified ORFs in the pangenome. The *pN*/*pS* ratio is defined as the ratio of the proportion of nonsynonymous polymorphisms to the proportion of synonymous polymorphisms given available sites. Excluding all nonpolymorphic ORFs and variants that do not pass the criteria (see Materials and Methods), 14,226 ORFs in the *Sulfurovum* pangenome had defined, finite *pN*/*pS* ratio values, with a mean of 0.1850. Most of the genes had *pN*/*pS* values of less than one, indicating purifying or stabilizing selection (Fig. 4A). In total, only 233 ORFs, 60 of which were singletons, had a *pN*/*pS* value over one with a maximum value of 3.1429, indicating positive selection. In contrast, 1,575 ORFs had a *pN*/*pS* value of 0, a strong negative selection signature. We observed different selective signatures across the *Sulfurovum* MAGs. Out of the 14,226 ORFs with a defined *pN*/*pS* value, only three of them were in Sulfurovum_01 and none in Sulfurovum_08. Additionally, Sulfurovum_16 and Sulfurovum_19 had ORFs with higher *pN*/*pS* values compared to the rest of the MAGs (Fig. S4A).

10.1128/mSystems.00673-19.4 FIG S4 *pN*/*pS* values for MAGs and ORFs in MAGs. (A) *pN*/*pS* values across MAGs, where the *pN*/*pS* ratios of each ORF is plotted in the boxplot as a function of the MAG it is found in. (B) *P* values for *pN*/*pS* ratio distribution. The pairwise Wilcoxon *P* values of *pN*/*pS* ratios (e.g., *pN*/*pS* ratios for *N* = 1 versus *N* > 1) as a function of pangenome frequency ares plotted. (C) *pN*/*pS* ratio across gene categories. The estimated *pN*/*pS* ratios of each identified ORF is plotted across each category of the ORF. (D) *pN*/*pS* values for phosphate-related genes. The estimated *pN*/*pS* ratio for each of the 21 phosphate uptake- and regulation-related ORFs is plotted on the bar graph. Download FIG S4, PDF file, 0.4 MB. Copyright © 2020 Moulana et al. 2020 Moulana et al. This content is distributed under the terms of the Creative Commons Attribution 4.0 International license.

Generally, genes that were more highly conserved across *Sulfurovum* MAGs had lower *pN*/*pS* values (Fig. 4A). For comparison, singletons had a mean *pN*/*pS* value of 0.2823, whereas genes found in all 22 MAGs had an average *pN*/*pS* value of 0.1244. These values significantly differed from the nonsingleton genes and genes not found in all MAGs, respectively (Fig. S4B; both *P* < 0.0001; Wilcoxon two-sided test). In addition, the mean *pN*/*pS* values for genes found in fewer than 20 MAGs (i.e., “accessory” genes) were significantly higher than for genes found in 22 MAGs (i.e., “core” genes) (Fig. 4B; *P* < 0.0001; Wilcoxon two-sided test). There was no correlation between the gene function category and the *pN*/*pS* ratios (Fig. S4C). We also observed that the *pN*/*pS* ratios for the phosphate uptake and regulation genes that were in higher abundance in Mid-Cayman Rise MAGs had an average *pN*/*pS* ratio of 0.0776 (Fig. S4D). Five of these 21 genes had a *pN*/*pS* ratio of 0.

**FIG 5:**
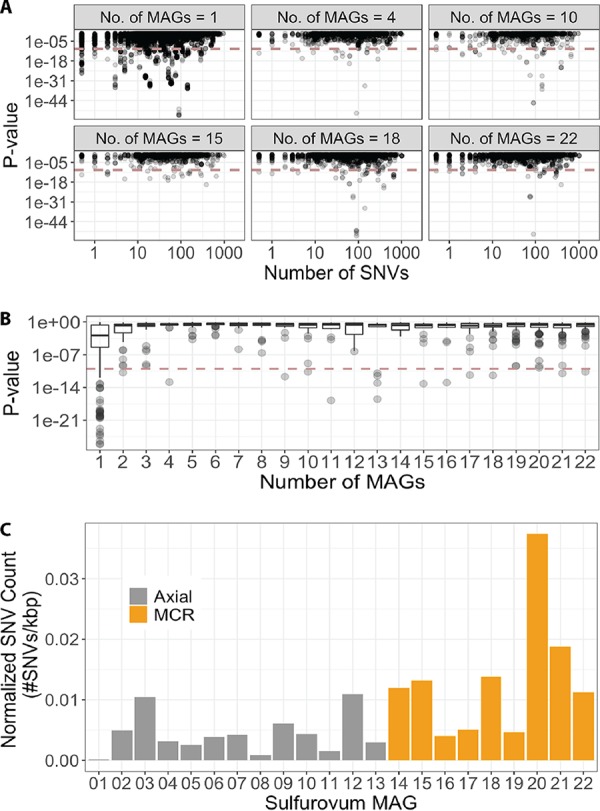
Gene-specific sweep signatures based on single nucleotide variants (SNVs) and *P* values. (A) The *P* value for each contig is plotted relative to the number of actual SNVs in the contig. Each point represents a single contig. The plots are separated according to the number of metagenome-assembled genomes (MAGs) in which that specific gene cluster was found. (B) *P* values as a function of gene cluster frequency in the pangenome. The *x* axis represents the number of MAGs sharing a specific gene cluster. (C) The SNV density for each MAG at either Axial Seamount of Mid-Cayman Rise. The MAG numbering is indicated in Table S2 in the supplemental material.

To determine whether the acquisition of specific genes led to gene-specific sweeps, we searched for unusually low single nucleotide variation (SNV) density contigs containing a gene in the pangenome. We assumed that the number of SNVs throughout the region follows a Poisson distribution with an expected value derived from the genome-wide SNV density (see Materials and Methods). Lower *P* values signify that the number of SNVs observed in the gene are lower than expected, indicating strong selective pressure. In general, singletons exhibited lower *P* values and a higher proportion of SNV-free contigs (Fig. 5A; Fig. S5A) compared to other genes. In fact, the singleton group had the highest proportion of genes with a *P* value of less than 1e−10 (Fig. S5B), whereas the gene clusters found in 22 MAGs (i.e., high-frequency genes) had the lowest proportion. When considering only genes in SNV-free contigs (1,382 genes are in SNV-free contigs), most of the genes with lower *P* values were singletons, which generally belonged to contigs with significantly lower SNV density (Fig. 5B). MAG Sulfurovum_01 from Axial Seamount had a particularly low SNV density of only 0.1 SNV per 1 kbp (Fig. 5C). Further, Sulfurovum_08 and Sulfurovum_11 from Axial Seamount had fewer than 2 SNVs per 1 kbp.

10.1128/mSystems.00673-19.5 FIG S5 Gene-specific sweeps vary based on pangenome frequency and SNV count. (A) For each pangenome frequency (the number of MAGs a gene is found in), the Poisson *P* value from the gene-specific sweep analysis is plotted against its SNV count. The dashed red line represents a *P* value of 1e−10, which is the cutoff in panel B. The point density in the plot is denoted by the darkness of the point (light to dark; low to high density). (B) Proportion of genes with sweep *P* value of <1e−10 across the number of MAGs. The proportion of ORFs with a Poisson *P* value of <1e−10 as a gene-specific sweep signature is plotted as a function of the pangenome frequency. Download FIG S5, PDF file, 0.4 MB. Copyright © 2020 Moulana et al. 2020 Moulana et al. This content is distributed under the terms of the Creative Commons Attribution 4.0 International license.

## DISCUSSION

Efforts to study wild microbial pangenomes face inherent challenges due to the difficulties of culturing environmental microorganisms (63). This study uses metagenomic data to investigate pangenome evolution in deep-sea hydrothermal vents, an environment where horizontal gene transfer, the primary mechanism of accessory genome accumulation, is extensive, and where microbial populations inhabit dynamic, geologically and geographically distinct habitats (38–41). We used metagenome-assembled genomes as an analog for genomes of a microbial population to study the forces that drive the evolution of microbial communities at deep-sea hydrothermal vents. To simplify our analyses, we focused on the pangenome of *Sulfurovum*, one of the most abundant genera in the two vent fields studied and one of the most important sulfur- and hydrogen-oxidizing bacteria found at hydrothermal vents globally (27, 34, 35, 50, 51) as well as in other sulfur-rich habitats, including caves and benthic sediments (64, 65).

### The *Sulfurovum* pangenome exhibits extensive variability and biogeographic structuring.

We observed extensive variation within the *Sulfurovum* pangenome, which is consistent with previous reports for this clade (51). Although some pairs of MAGs had high (>90%) overlap in gene content, most MAG pairs did not share a high proportion of gene content. In fact, genes found in all 22 MAGs made up less than 5% of the whole pangenome. This is possibly an artifact of the incomplete nature of MAGs, such that some genes that appear to be accessory actually belong to the core genome. However, the low number of universally conserved genes has also previously been observed in the pangenomes of Escherichia coli (13%) at the species level (66) and in the low-light *Prochlorococcus* clade (10%) at the genus level (67). Similarly, as more MAGs are recovered and more *Sulfurovum* genomes are sequenced, we expect the size of the core genome to continue to decrease in size (see Fig. S2A in the supplemental material), as is commonly observed in pangenomic studies (7).

To understand the evolutionary mechanisms that led to this observed variation, we identified the gene functions encoded by the *Sulfurovum* MAGs. The gene function analyses suggested that translation, coenzyme metabolism, amino acid metabolism, lipid metabolism, and cell cycle functions were all enriched among higher-frequency genes. Cells need fundamental housekeeping genes to function, necessitating the maintenance of such genes across genomes and ensuring a low proportion of these genes being lost. This maintenance of housekeeping cell cycle functions in a core genome is frequently observed in pangenome studies (63, 68). The maintenance of some housekeeping genes at medium or low frequency might be a result of the various translational and housekeeping complexes that are only partially conserved across genomes. In addition, we observed a high proportion of signal transduction, membrane/envelope biogenesis, and carbohydrate metabolism functions in lower-frequency genes, while these functions were rare in higher-frequency genes. Moreover, ion transport and metabolism functions were especially enriched in medium-frequency genes. The presence of nutrient uptake and membrane-related genes in the variable genome has been documented previously in nonvent environments (68–70) as well as in the Epsilonbacteraeota genus *Lebetimonas* from hydrothermal vent systems (17). Cell membrane proteins represent the first contact between the cell and the environment and are also common binding sites for viruses, and thus, diversifying selection may operate on these genes in response to environmental stimuli. The nonrandom distribution of different functions according to gene frequency suggests that the accumulation of genes in the pangenome is likely maintained by a frequency-dependent selection mechanism that suppresses the frequency of some genes while allowing the spread and conservation of other genes (24, 25, 32).

Importantly, we also observed biogeographic structuring of the *Sulfurovum* pangenome. Biogeographic structuring of microbial communities has previously been observed at the community level in deep-sea hydrothermal vent systems, with divergent community structure persisting over time within individual vent sites (34, 58, 59). This most likely results from the subseafloor plumbing at individual sites and the degree of fluid flux between sites, which may isolate communities and produce divergent population structure within hydrothermal systems, even at the scale of individual vents (71). However, biogeographic studies have not been conducted at the population or pangenomic scale for microbial populations in hydrothermal systems. Here, we found some evidence for biogeographic structuring in the *Sulfurovum* pangenome, with more migration occurring locally. Some Axial Seamount *Sulfurovum* MAGs tended to cluster together with regard to their gene content similarity, as did the Mid-Cayman Rise MAGs. However, the MAGs did not cluster strictly by geographic location (Fig. 1), and there was high local gene content diversity within each region. Although different environments have been shown to harbor closely related microbial populations with divergent gene content due to selection (68, 72, 73), this pattern suggests that the clustering is more likely to be the result of a relaxed migration barrier within the local vent field compared to the barrier between the ocean basins. This weak biogeographic structuring within vent fields suggests that gene flow is not restricted among distinct *Sulfurovum* populations within vent fields at the evolutionary scales that mold a pangenome. It is important to note that the 22 *Sulfurovum* MAGs analyzed here do not represent an exhaustive data set, and therefore, most likely, some amount of genomic variation is missed. Nevertheless, these data sets were sufficient to observe large-scale biogeographic patterns between and within ocean basins.

We propose that natural selection acting on the *Sulfurovum* pangenome leads to local adaptation of microbial populations to their respective vent environment. The genome similarity analyses revealed gene content differences between Axial Seamount and Mid-Cayman Rise *Sulfurovum* MAGs, in which some genes were more enriched in one vent field compared to the other. The clearest and most striking example of selection molding the *Sulfurovum* pangenome emerges from patterns regarding phosphate uptake. Phosphate regulation- and uptake-related genes were enriched in Mid-Cayman Rise *Sulfurovum* MAGs compared to Axial Seamount MAGs. This observed pattern most likely results from the lower phosphate content of the Atlantic Ocean, where the Mid-Cayman Rise is located, relative to that of the Pacific Ocean, where Axial Seamount is located (74–76). This result is consistent with a previous *Prochlorococcus* study in the surface ocean showing enrichment of phosphate-acquisition genes in *Prochlorococcus* strains in the Atlantic compared to the Pacific (73). Deep-water phosphate values are approximately 1 mm/kg in the Atlantic Ocean and 3 mm/kg in the Pacific Ocean (74), and end-member hydrothermal fluid has extremely low phosphate concentrations because phosphate is removed by hydrothermal processes (77). The diffuse fluids in which hydrothermal microbial communities are found represent a mixture of deep seawater and hydrothermal fluid, and our results indicate that the differences in nutrient abundances that distinguish the Atlantic and Pacific Oceans are strong enough to mold the genomes of microbes inhabiting hydrothermal vent systems. Moreover, arsenate reductase genes were more highly represented in the Mid-Cayman Rise *Sulfurovum* MAGs. This may result from phosphate scavenging in a phosphate-limited environment, which can cause microorganisms to take up arsenate instead, requiring arsenate reduction into arsenite by arsenate reductase in order to ameliorate arsenate toxicity (73, 78). Because arsenic is known to be naturally enriched in deep-sea hydrothermal systems (79, 80), this arsenate reduction mechanism is particularly important in these habitats, especially when phosphate is limited. Our results extend the range of phosphate- and arsenate-driven selection pressures from the surface oceans (68, 72, 73) to the extreme deep-sea environment despite the multifold increase in phosphate content from the surface to the deep ocean (74, 75).

### Signatures of selection in the *Sulfurovum* pangenome.

If natural selection drives the accumulation of genes in the accessory genome, then evolution should act differently on the accessory genes compared to the core genome. Newly acquired genes might be expected to first undergo positive selection before purifying selection. For instance, Mid-Cayman Rise microbes are adapted to the phosphate-limited environment by incorporating phosphate uptake and regulation genes. These genes exhibited purifying selection with near-zero *pN*/*pS* ratio (Fig. S4D), suggesting that they are already adaptive. In contrast, other accessory genes can have a very different evolutionary scheme. When these genes are beneficial but not yet at the peak of their fitness landscape, positive selection would be expected to act on them. As a result, some accessory genes might have a low *pN*/*pS* ratio due to local adaptation or frequency-dependent adaptation, but some have a higher *pN*/*pS* ratio due to positive selection. In accordance with this, our results show that the *pN*/*pS* ratio spread among lower-frequency genes was higher than that of higher-frequency genes (Fig. 4 and Fig. S6). Moreover, lower-frequency genes in the *Sulfurovum* pangenome generally had a higher *pN*/*pS* ratio compared to the higher-frequency genes, suggesting that genes undergoing positive selection were more likely to be accessory genes than core genes. While it is difficult to determine absolutely from the *pN*/*pS* ratio whether a gene is undergoing positive or negative selection (81), previous work has found that accessory genes undergo more relaxed purifying or negative selection compared to core genes in bacterial genomes (82, 83) or stronger positive selection in the case of Pseudomonas aeruginosa populations (84). Thus, our results are consistent with previous work and suggest that selective pressures on the higher- and lower-frequency genes differ, where the lower-frequency genes are more likely to be under positive selection.

10.1128/mSystems.00673-19.6 FIG S6 Standard deviations of *pN*/*pS* for ORFs according to the number of MAGs that ORF is found in. The standard deviations of *pN*/*pS* ratios as a function of pangenome frequency are plotted. Download FIG S6, PDF file, 0.1 MB. Copyright © 2020 Moulana et al. 2020 Moulana et al. This content is distributed under the terms of the Creative Commons Attribution 4.0 International license.

Moreover, the ecotype model proposed by Cohan et al. (85) suggests that an individual gaining an evolutionarily beneficial trait should have higher fitness than others in the population, causing it to sweep the population and thus purging genomic diversity. “Ecotype” here is defined as a group of ecologically similar bacteria in which genetic diversity is limited due to selection, drift, or both. However, evidence for genome-wide selective sweeps have mainly come from theoretical simulations and laboratory experiments, whereas naturally occurring sweeps have rarely been observed (23). In the two hydrothermal systems, we observed a significantly low SNV density in MAG Sulfurovum_01 from one vent at Axial Seamount, possibly resulting from either a selective sweep or clonal expansion (Fig. 5C). Purging variants from the population via a sweep or rapid growth of a single clone would reduce nucleotide diversity in the population, resulting in low SNV density. Other MAGs, such as Sulfurovum_08 and Sulfurovum_11 from Axial Seamount, also harbored this low sequence diversity. These Sulfurovum MAGs had lower SNV density than the Sulfurovum MAGs previously reported from the Mid-Cayman Rise that may have been undergoing selective sweeps or clonal expansions (27). Without time-series data. we cannot distinguish whether this low diversity was caused by a recent bloom event or a selective sweep. However, previous work at Axial Seamount observed an increase in the abundance of *Sulfurovum* at Marker 113 in 2014 compared to other years (58), and Sulfurovum_08 was recovered from this sample, supporting the hypothesis that a clonal expansion was responsible for the reduced sequence diversity in this MAG.

When the recombination rate and gene exchange among individuals is high enough, specific genes could sweep throughout populations independently of the rest of the genome (29, 31). This gene-specific sweep could also be promoted by phage predation that causes negative frequency-dependent selection (86). Gene-specific sweeps are likely to be particularly important in hydrothermal vent systems due to the high rate of horizontal gene transfer within and among microbial populations in this system (43). This high HGT rate not only creates highly diverse pangenomes but also allows for selection to act on a gene-by-gene basis. We indeed observed that specific regions of the *Sulfurovum* MAGs had lower SNV density compared to the rest of the genome, especially for regions containing singleton genes (Fig. 5). Not only were singletons more likely to have lower SNV density compared to the rest of the genome, they were more likely to be SNV free. Although SNV-free regions could be pervasive, especially in low-SNV-density genomes such as Sulfurovum_01, SNV-free singletons were contained in high-SNV-density genomes too. One possible reason for this observation is low coverage of singletons compared to other genes, but we found no evidence of such bioinformatic artifacts (Fig. S7). Assuming no other possible bioinformatic artifact that results in this observation, gene-specific sweeps might occur more frequently in singletons than in other genes. In this case, the evidence suggests that gene-specific sweeps mostly occur in genes that are newly acquired in the species.

10.1128/mSystems.00673-19.7 FIG S7 Average coverage of contigs across the number of MAGs. The average coverage of each contig an ORF is found in is plotted against the number of MAGs that ORF is found in. Download FIG S7, PDF file, 0.1 MB. Copyright © 2020 Moulana et al. 2020 Moulana et al. This content is distributed under the terms of the Creative Commons Attribution 4.0 International license.

Investigating patterns of selection in microbial genomes is crucial for understanding how microbial populations evolve and adapt to the environment over time. Here, we show evidence for natural selection operating on the *Sulfurovum* pangenome of hydrothermal vents and identify some of the drivers of that selection. Genes that are highly conserved in the *Sulfurovum* pangenome have different functional annotations than lower-frequency genes, suggesting that gene acquisition is not random. At the Mid-Cayman Rise, *Sulfurovum* populations appear to have adapted to the low-phosphate environment through the acquisition and maintenance of phosphate uptake and regulation-related genes. In addition, *pN*/*pS* ratios reveal that lower-frequency genes are either more susceptible to positive selection or more resistant to negative selection than higher-frequency genes. Finally, we observed some evidence for a genome-wide sweep in one of the *Sulfurovum* populations and gene-specific sweep events in singleton genes. Altogether, we have revealed patterns in the pangenome structure of *Sulfurovum* populations from two distinct hydrothermal vent regions and conclude that their accessory genome structure is molded by natural selection rather than neutral forces. These analyses of genomic variation provide important insights into the dynamics that drive diversity and mold the evolution of microbial populations.

## MATERIALS AND METHODS

### Data collection.

All of the data used for this study was collected from samples obtained from diffuse flow hydrothermal vent fluids emanating from seafloor rocks and sulfide deposits. All methods for sample collection, DNA extraction, and sequencing are described by Reveillaud et al. (50) and Fortunato et al. (58) for Axial Seamount and by Reveillaud et al. (50) and Anderson et al. (27) for the Mid-Cayman Rise. For all samples, diffuse flow hydrothermal fluid was filtered through 0.2-μm filters *in situ* while monitoring temperature to capture the microbial communities. We stored filters at −80°C onboard the ship and extracted DNA on shore as described in the above publications. All samples were sequenced at the Josephine Bay Paul Center at the Marine Biological Laboratory using the Illumina HiSeq or NextSeq sequencing platform. These metagenomes were previously described by Fortunato et al. (58), Reveillaud et al. (50), Anderson et al. (27), and Galambos et al. (54). The data are available from the European Nucleotide Archive Archive (ENA) under study accession numbers PRJEB7866, PRJEB12000, and PRJEB19456 for 2013, 2014, and 2015, respectively, at Axial Seamount, and under study accession PRJEB15541 for the Mid-Cayman Rise. All metagenomes used for this study are shown in Table S1 in the supplemental material.

10.1128/mSystems.00673-19.8 TABLE S1 All metagenomic samples included in this study. Download Table S1, DOCX file, 0.01 MB. Copyright © 2020 Moulana et al. 2020 Moulana et al. This content is distributed under the terms of the Creative Commons Attribution 4.0 International license.

10.1128/mSystems.00673-19.9 TABLE S2 All *Sulfurovum* MAGs. Each *Sulfurovum* MAG recovered from the two hydrothermal sites is listed with its ID, number of contigs, length, GC content, completion, redundancy, and number of genes present. Download Table S2, DOCX file, 0.02 MB. Copyright © 2020 Moulana et al. 2020 Moulana et al. This content is distributed under the terms of the Creative Commons Attribution 4.0 International license.

### Metagenome assembly and mapping.

We conducted all metagenomic processing of *Sulfurovum* MAGs from the Mid-Cayman Rise samples as described by Anderson et al. (27). Briefly, we quality filtered all reads using the illumina-utils package (87), assembled with idba-ud v.1.1.2 (88), and reads were mapped to contigs using bowtie v1.2.2 (89). For the Axial Seamount metagenomes, we first quality filtered the reads using “iu-filter-quality-minoche” within the illumina-utils package (87) and then assembled metagenomic reads using idba-ud v1.1.3 (88) with default settings. For subsequent analyses, we included only contigs of at least 1,000 bp in length to ensure robust contig clustering based on tetranucleotide frequency and coverage. We mapped the metagenomic reads of each sample to the assembled contigs using bowtie v1.2.2 (89) with default settings. We used anvi’o v4.1.0 (90) to organize the metagenomic contig samples into profiles using the anvi’o command “anvi-profile” with the flag “--profile-SCVs” in order to detect and analyze single nucleotide variants (SNVs) and single codon variants (SCVs).

### Metagenome binning and gene annotation.

We created metagenomic bins based on tetranucleotide composition and relative coverage of each contig across all samples using anvi’o. To estimate the completion and redundancy of metagenomic bins, anvi’o used PRODIGAL v2.6.3 (91) to identify open reading frames (ORFs) in our contigs, and HMMER v3.1b2 (92) to search for the presence of single-copy core genes in bins based on collections for bacteria (93) and archaea (94). Using these estimates, we designated 159 metagenomic bins as metagenome-assembled genomes (MAGs) using a threshold of <10% redundancy and >70% completion, where “redundancy” is determined by the identification of multiple copies of genes that are usually present in a single copy within microbial genomes. We determined the taxonomy of each MAG using PhyloSift (95), using the “phylosift all” flag. Bins that were characterized by PhyloSift as having multiple significant taxonomic hits were excluded from the analysis. We identified 13 *Sulfurovum* MAGs from the Axial Seamount samples and 8 *Sulfurovum* MAGs from the Mid-Cayman Rise samples.

### Tree construction.

The *Sulfurovum* phylogenomic tree was constructed by compiling aligned, concatenated single-copy universal genes created by PhyloSift (95) (using the concat.codon.updated.1.fasta output) for all *Sulfurovum* MAGs in addition to seven reference genomes (NCBI taxonomy identifiers [NCBI:txid] are shown in parentheses): Sulfurimonas denitrificans DSM1251 (NCBI:txid326298), *Sulfurovum* sp. strain PC08-66 (NCBI:txid1539063), *Sulfurovum* sp. strain FS08-3 (NCBI:txid1539065), *Sulfurovum* sp. strain AS07-7 (NCBI:txid1539062), *Sulfurovum* sp. strain FS06-10 (NCBI:txid1539064), *Sulfurovum* sp. strain SCGC AAA036-F05 (NCBI:txid1218800), and Sulfurovum lithotrophicum strain ATCC BAA-797 (NCBI:txid206403). The tree was constructed using RAxML v.8.2.9 (96) with 100 rapid bootstraps and the GTRGAMMA model (general tree reversible model with gamma distribution) of rate heterogeneity, using Sulfurimonas denitrificans DSM1251 as the outgroup.

### Pangenome profiling.

We used a pangenomic workflow within anvi’o to build and perform analyses on the *Sulfurovum* pangenome (67). First, using anvi’o, we constructed a genome storage database that stores all reads, contigs, nucleotide variation, and annotation information from all *Sulfurovum* MAGs. Then, using the “anvi-pan-genome” command, we annotated the called ORFs in each MAG with Diamond v0.9.22.123 (97) to compare each ORF against NCBI’s Conserved Domains Database to obtain the cluster of orthologous groups (COG) annotation for each ORF with a maximum E value of 1e−05. Anvi’o then resolved gene clusters across all MAGs using a Markov Cluster Algorithm (MCL) (98) with default arguments (minbit value of 0.5 and inflation value of 2) using the minbit heuristic implemented from ITEP to eliminate weak amino acid matches so that only ORFs with strong amino acid similarities were clustered together (90, 97). The gene clusters were sorted based on their frequency across MAGs to create an across-MAG gene presence-absence matrix. We used the gene presence-absence matrix to conduct a pairwise comparison of the MAGs and draw their distance based on the number of genes present in a MAG that were not contained in another MAG, from which we created a hierarchical clustering dendrogram in R (99). In order to take into account the various levels of MAG completion, we estimated the probability for a gene cluster found in *n* MAGs to be found in all MAGs for *n < *22 by independently simulating the probability of some genes to be missing in a MAG (scripts and an explanation of the code provided at https://github.com/carleton-spacehogs/pangenome-selection).

### Gene annotation analyses.

For all analyses on gene functions, we used the annotations produced in the pangenome profiling step implemented in anvi’o. Although most gene clusters mapped to a unique annotation, a small proportion did not. In those cases, we assigned multiple annotations for those clusters. We then conducted analysis on these annotations and their COG categories in R (see the above URL for the GitHub page for the code and a description of the code). Moreover, we studied the enrichment of some genes in one specific vent environment compared to the other (e.g., Axial Seamount compared to Mid-Cayman Rise) by assuming that the number of Axial genomes in which a gene is found in follows *Binom(N*, *p)* where *N* is the total number of genomes the gene is found in and *P* = 13/22.

### *pN*/*pS* ratio and sweep analyses.

We used anvi’o (90) to conduct a gene-by-gene *pN*/*pS* ratio analysis using single codon variation (SCV), single nucleotide variation (SNV), and single amino acid variation (SAAV) counts. We generated these counts using the anvi’o command “anvi-gen-variability-profile” for each of the *Sulfurovum* MAGs with the “--engine CDN” flag to obtain SCV variability profiles and the “--engine AA” to obtain SAAV profiles. We ran the anvi’o python script “anvi-script-calculate-pn-ps-ratio” to determine the *pN*/*pS* ratio, which calculates the number of synonymous and nonsynonymous variants, normalized by the potential number of synonymous and nonsynonymous variants. Only reads that cover the full codon context were used for variant detection in this analysis. We analyzed the distribution of SNVs across the pangenome using the variability profile generated with the default option for flag “--engine”. We first counted the number of SNVs in each MAG (see the above URL for the GitHub page for the code and an explanation of the code) and then calculated the number of SNVs in the contig in which each ORF was found. We assumed that the number of SNVs in the contig region follows the distribution *Poiss(n_c_)* where *n_c_* is the expected value of SNVs in a contig *c* for *n_c_ = N l_c_*/*L*. *N* is the total number of SNVs in the MAG, *L* is the length of the MAG, and *l_c_* is the length of contig *c*. We computed the Poisson cumulative distribution function (CDF) value for each gene and ascribed the value as the gene-specific sweep *P* value for the gene.

### Data and script accessibility.

All R, Python scripts, an explanation of code, and raw results used for this analysis are publicly available on GitHub at https://github.com/carleton-spacehogs/pangenome-selection.
