# Supplementary figures and images for: Selection Is a Significant Driver of Gene Gain and Loss in the Pangenome of the Bacterial Genus Sulfurovum in Geographically Distinct Deep-Sea Hydrothermal Vents
Source: mSystems. 2020 Apr 14;5(2):e00673-19. doi: 10.1128/mSystems.00673-19 (PMC7159903; doi:10.1128/mSystems.00673-19)

A)

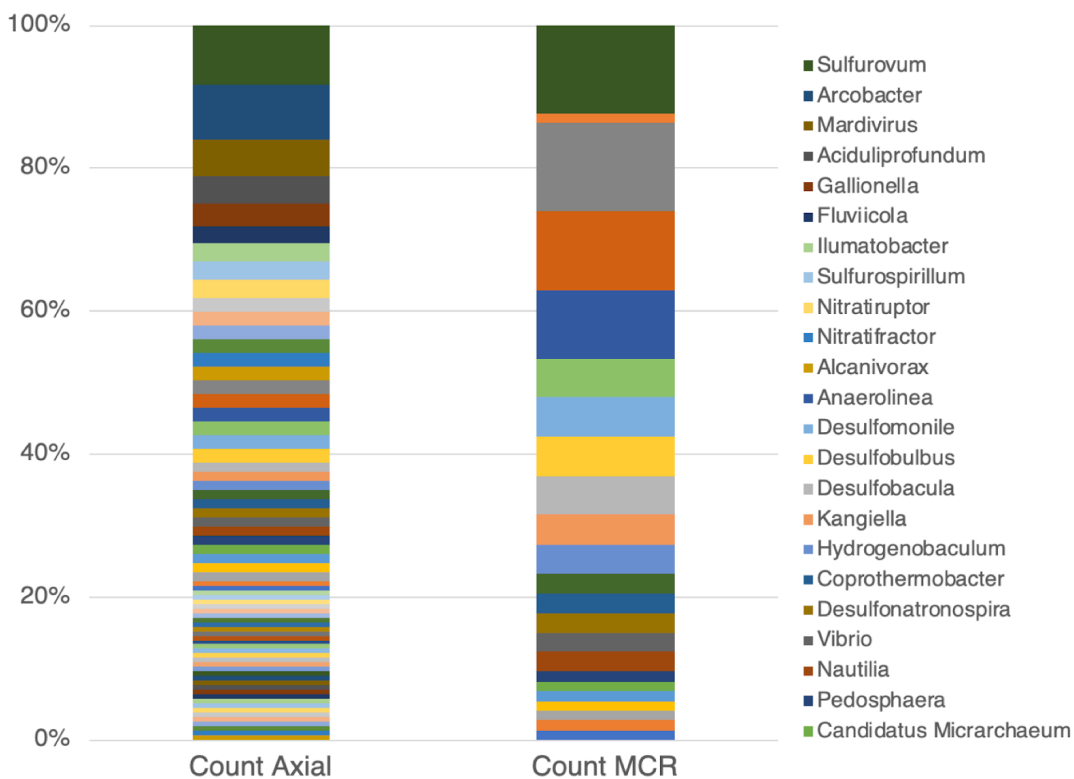

B)

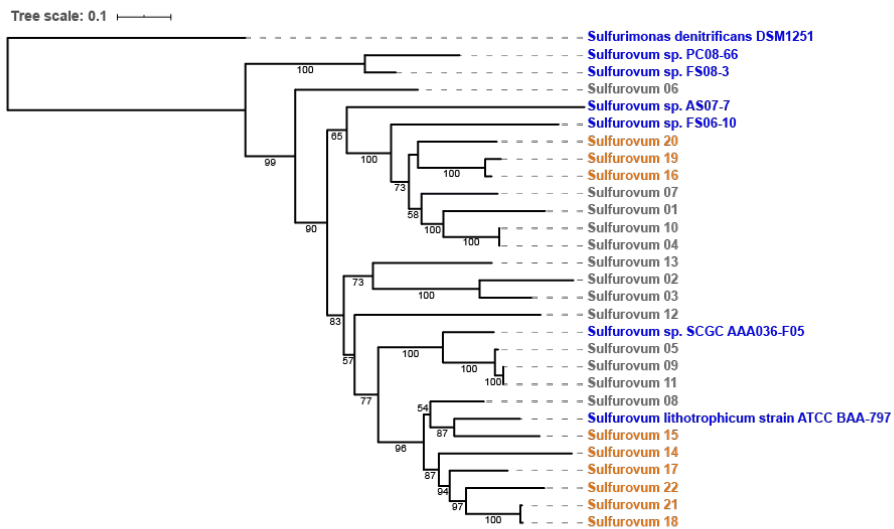

Supplement: FIG S1 [file mSystems.00673-19-sf001.pdf]

**A)**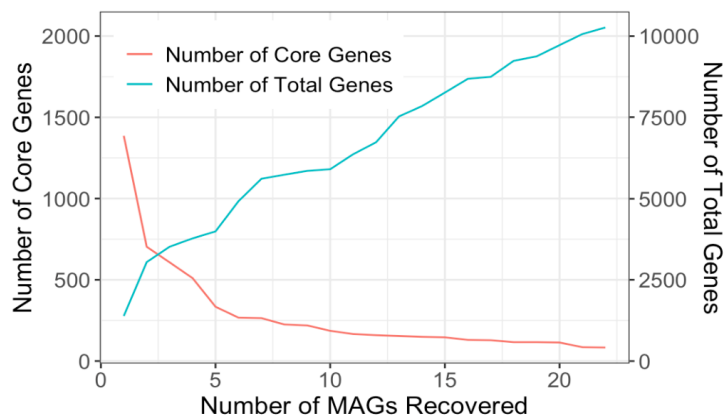**B)**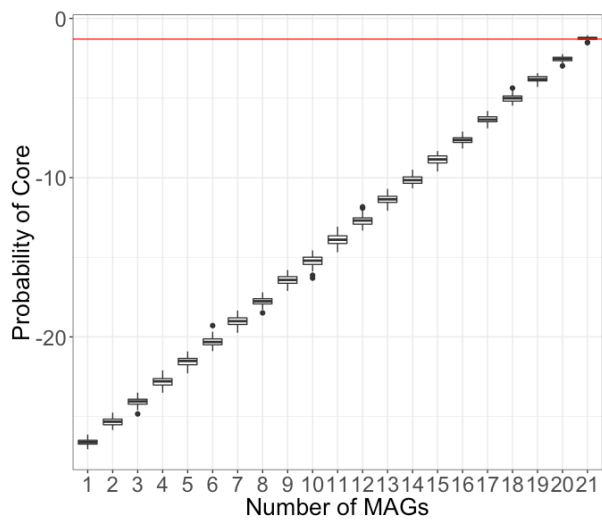

Supplement: FIG S2 [file mSystems.00673-19-sf002.pdf]

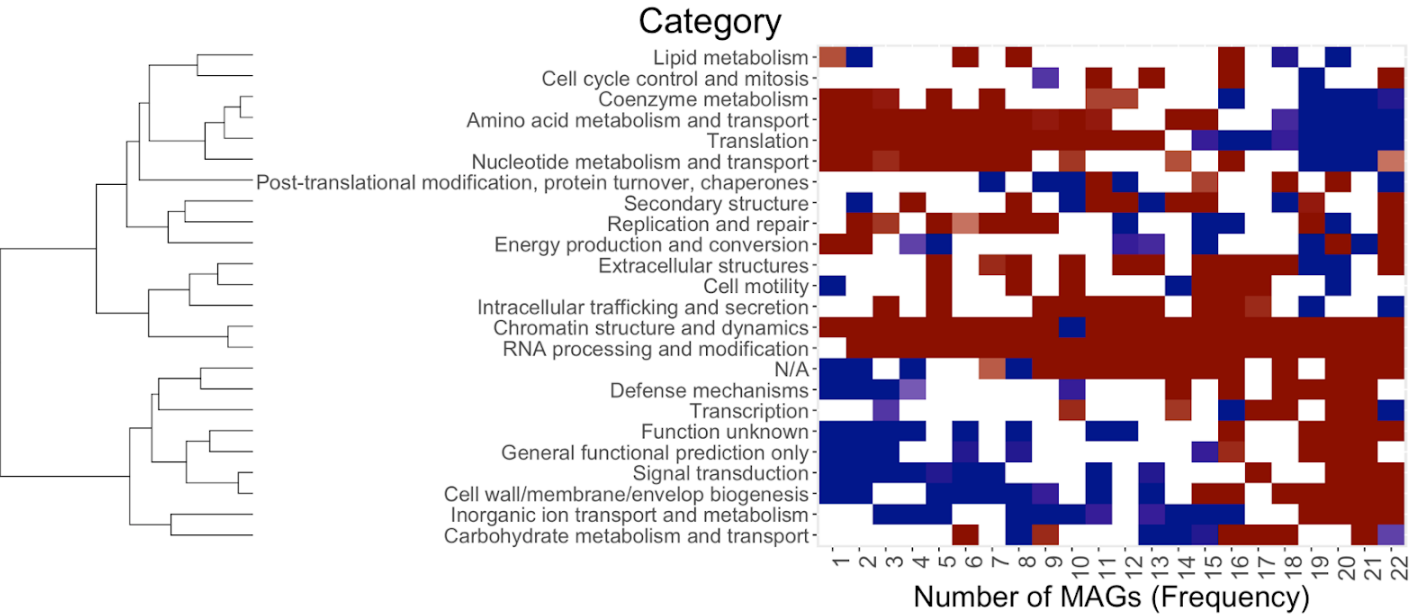

Supplement: FIG S3 [file mSystems.00673-19-sf003.pdf]

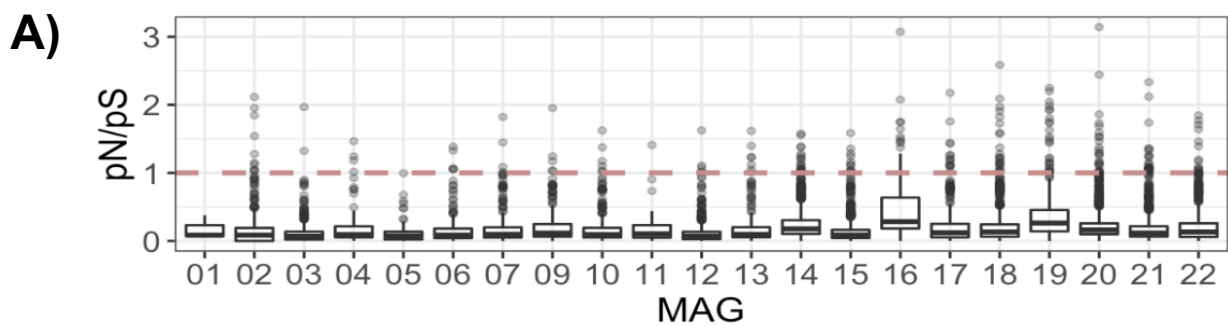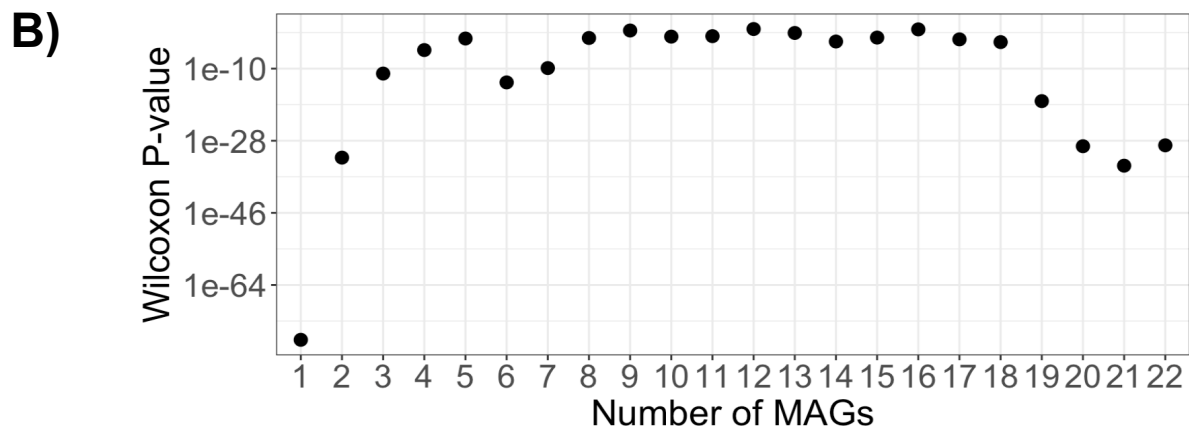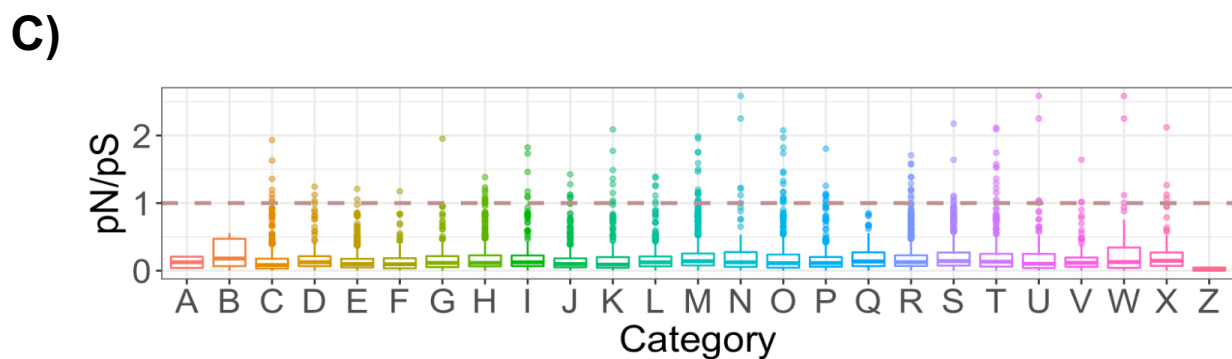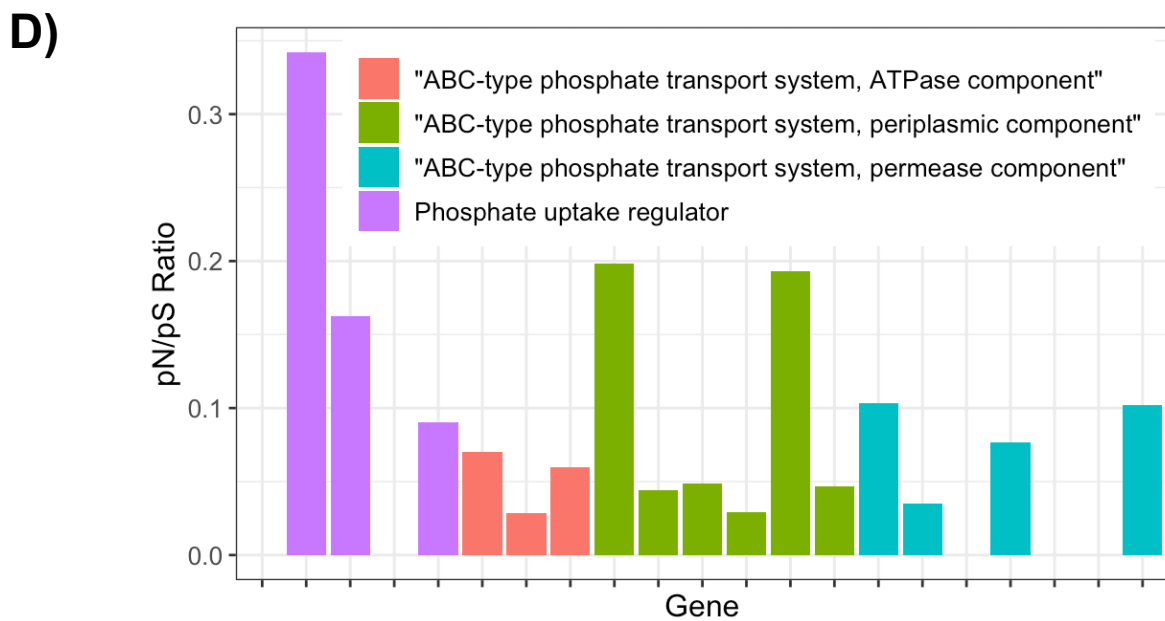

Supplement: FIG S4 [file mSystems.00673-19-sf004.pdf]

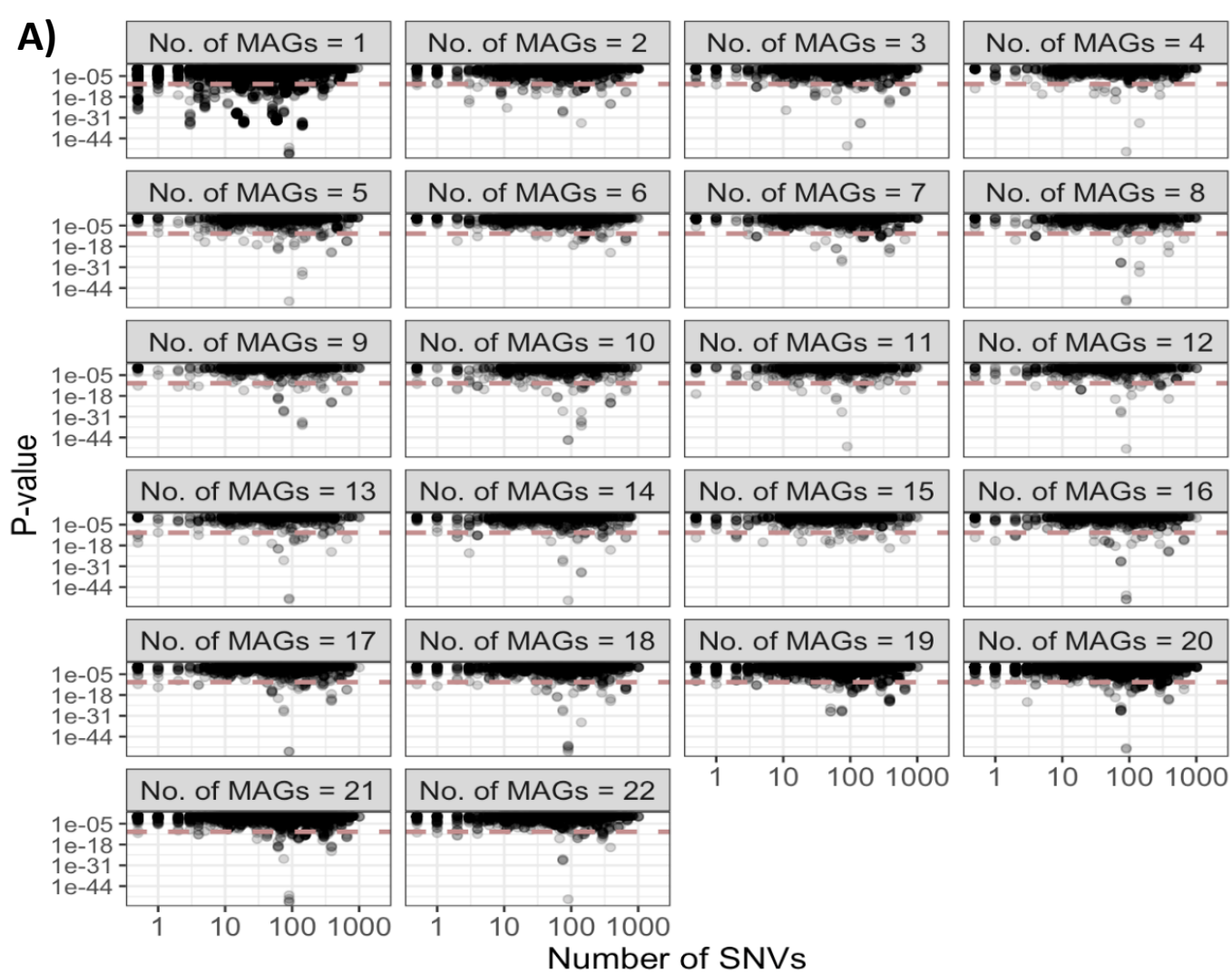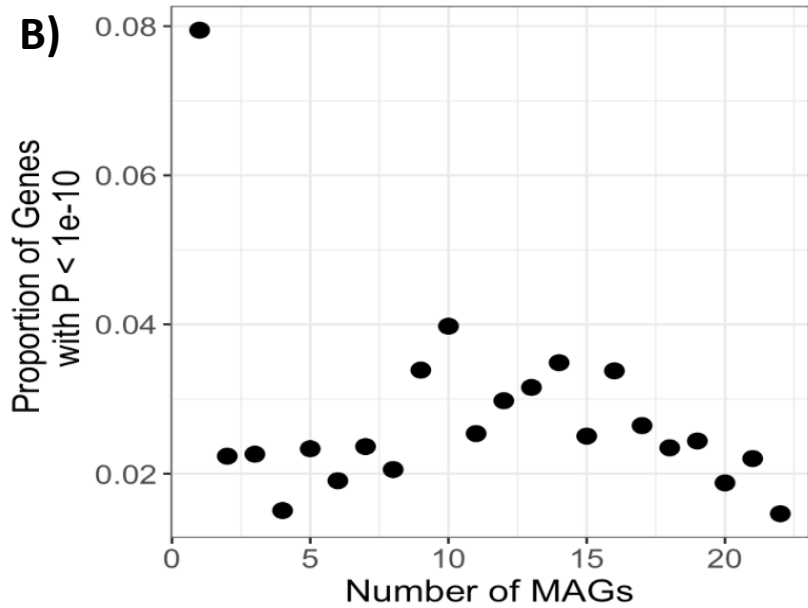

Supplement: FIG S5 [file mSystems.00673-19-sf005.pdf]

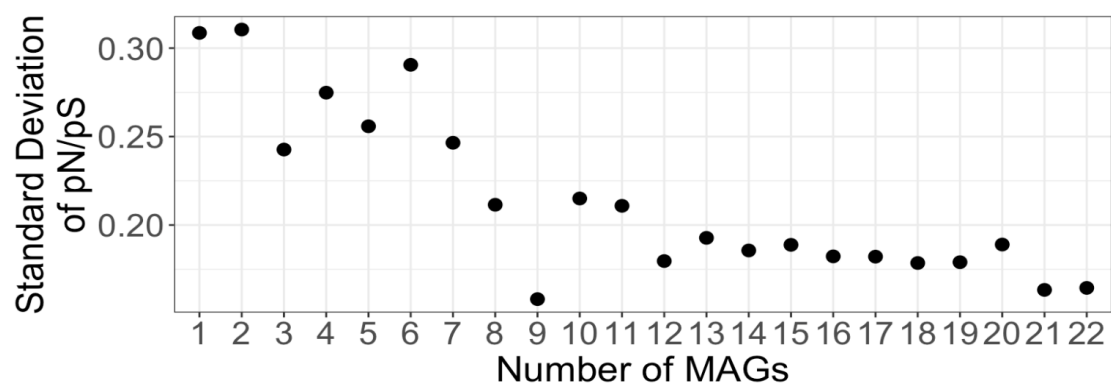

Supplement: FIG S6 [file mSystems.00673-19-sf006.pdf]

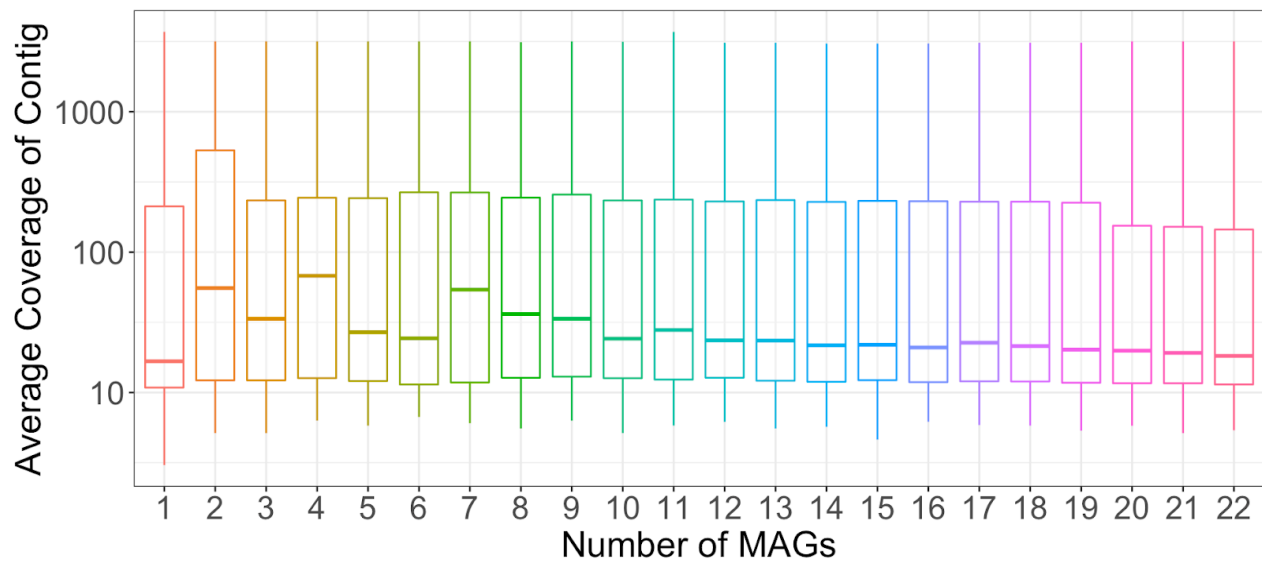

Supplement: FIG S7 [file mSystems.00673-19-sf007.pdf]
