# Supplementary material for: Selection Is a Significant Driver of Gene Gain and Loss in the Pangenome of the Bacterial Genus Sulfurovum in Geographically Distinct Deep-Sea Hydrothermal Vents
Source: mSystems. 2020 Apr 14;5(2):e00673-19. doi: 10.1128/mSystems.00673-19 (PMC7159903; doi:10.1128/mSystems.00673-19)
Supplement: TABLE S1 [file mSystems.00673-19-st001.docx]

| **Location** | **Vent Field** | **Vent** | **Sample** | **Year Sampled** | **Max Temp. (˚C)** | **ENA Secondary Accession** | **ENA Sample Accession** |
| --- | --- | --- | --- | --- | --- | --- | --- |
| MCR | Von Damm | Shrimp Hole | FS844 | 2012 | 50 | ERS1370015 | SAMEA4470836 |
| MCR | Piccard | X-19 at BV 4, BVM | FS854 | 2012 | 18 | ERS1370020 | SAMEA4470841 |
| MCR | Piccard | Shrimp Gulley 2, BSM | FS856 | 2012 | 108 | ERS1370021 | SAMEA4470842 |
| MCR | Von Damm | near Main Orifice | FS866 | 2013 | 130 | ERS1370022 | SAMEA4470843 |
| MCR | Von Damm | Old Man Tree | FS881 | 2013 | 114 | ERS1370027 | SAMEA4470848 |
| Axial | International District | El Guapo | FS896 | 2013 | 25.9 | ERS614329 | SAMEA3143269 |
| Axial | SE Rift Zone | N3 Area | FS898 | 2013 | 20.1 | ERS614331 | SAMEA3143271 |
| Axial | Outside Caldera | Dependable | FS900 | 2013 | 53.3 | ERS614333 | SAMEA3143273 |
| Axial | SE Caldera | Marker 113 | FS903 | 2013 | 24.7 | ERS614323 | SAMEA3143263 |
| Axial | SE Rift Zone | Marker 33 | FS904 | 2013 | 27.6 | ERS614325 | SAMEA3143265 |
| Axial | SE Caldera | Marker 113 | FS906 | 2014 | 25.6 | ERS1003595 | SAMEA3696446 |
| Axial | SE Rift Zone | Marker 33 | FS908 | 2014 | 20.7 | ERS1003599 | SAMEA3696450 |
| Axial | SE Rift Zone | Marker 33 | FS917 | 2015 | 40.9 | ERS1573120 | SAMEA101254168 |

**Supplementary Table 1.** All metagenomic samples included in this study.
