# Supplementary material for: Selection Is a Significant Driver of Gene Gain and Loss in the Pangenome of the Bacterial Genus Sulfurovum in Geographically Distinct Deep-Sea Hydrothermal Vents
Source: mSystems. 2020 Apr 14;5(2):e00673-19. doi: 10.1128/mSystems.00673-19 (PMC7159903; doi:10.1128/mSystems.00673-19)
Supplement: TABLE S2 [file mSystems.00673-19-st002.docx]

| **Loc-ation** | **Vent Field** | **Vent** | **Sample** | **Bin** | **ID** | **Length** | **# Contigs** | **N50** | **GC** | **Completion** | **Redun-dancy** | **Genome** | **# Genes** |
| --- | --- | --- | --- | --- | --- | --- | --- | --- | --- | --- | --- | --- | --- |
| Axial | Dependable | Outside Caldera | FS900 | Bin_15 | FS900-Bin_15 | 1427644 | 182 | 9601 | 31.1838 | 71.9424 | 1.4388 | Sulfurovum_01 | 1444 |
| Axial | El Guapo | International District | FS896 | Bin_8 | FS896-Bin_8 | 2206642 | 249 | 12292 | 50.5738 | 88.4892 | 2.8777 | Sulfurovum_02 | 2470 |
| Axial | El Guapo | International District | FS896 | Bin_9 | FS896-Bin_9 | 1790541 | 339 | 5975 | 46.8953 | 86.3309 | 6.4748 | Sulfurovum_03 | 2001 |
| Axial | El Guapo | International District | FS896 | Bin_4 | FS896-Bin_4 | 1334830 | 245 | 5917 | 30.7604 | 76.2590 | 2.1583 | Sulfurovum_04 | 1431 |
| Axial | Marker 33 | SE Rift Zone | FS908 | Bin_1 | FS908-Bin_1 | 875077 | 177 | 5067 | 35.6743 | 70.5036 | 0.7194 | Sulfurovum_05 | 980 |
| Axial | Marker 33 | SE Rift Zone | FS917 | Bin_6 | FS917-Bin_6 | 1140286 | 237 | 4885 | 29.7957 | 84.1727 | 4.3165 | Sulfurovum_06 | 1269 |
| Axial | Marker 113 | SE Caldera | FS906 | Bin_8 | FS906-Bin_8 | 2048994 | 405 | 5498 | 35.7572 | 71.2230 | 2.1583 | Sulfurovum_07 | 2152 |
| Axial | Marker 113 | SE Caldera | FS906 | Bin_6 | FS906-Bin_6 | 2084665 | 168 | 17046 | 37.9433 | 84.1727 | 2.1583 | Sulfurovum_08 | 2169 |
| Axial | Marker 113 | SE Caldera | FS906 | Bin_12 | FS906-Bin_12 | 1042616 | 119 | 10427 | 35.1747 | 91.3669 | 0.7194 | Sulfurovum_09 | 1133 |
| Axial | Marker 33 | SE Rift Zone | FS904 | Bin_4 | FS904-Bin_4 | 1747597 | 168 | 14127 | 30.4161 | 94.2446 | 3.5971 | Sulfurovum_10 | 1777 |
| Axial | Marker 113 | SE Caldera | FS903 | Bin_5 | FS903-Bin_5 | 894463 | 168 | 5666 | 35.1039 | 83.4532 | 2.1583 | Sulfurovum_11 | 1031 |
| Axial | N3 Area | SE Rift Zone | FS898 | Bin_12 | FS898-Bin_12 | 1550985 | 163 | 12047 | 36.2053 | 96.4029 | 0.7194 | Sulfurovum_12 | 1614 |
| Axial | N3 Area | SE Rift Zone | FS898 | Bin_1 | FS898-Bin_1 | 1591765 | 164 | 11987 | 34.0510 | 96.4029 | 0.7194 | Sulfurovum_13 | 1674 |
| MCR | Von Damm | Shrimp Hole | FS844 | Bin_43 | FS844-Bin_43 | 1918793 | 239 | 10177 | 34.5760 | 97.1223 | 1.4388 | Sulfurovum_14 | 2135 |
| MCR | Von Damm | Shrimp Hole | FS844 | Bin_13 | FS844-Bin_13 | 1767871 | 212 | 12779 | 38.5437 | 92.8058 | 5.7554 | Sulfurovum_15 | 1908 |
| MCR | Piccard | X-19 at BV 4, BVM | FS854 | Bin_99 | FS854-Bin_99 | 1870573 | 152 | 18565 | 35.7590 | 92.0863 | 2.1583 | Sulfurovum_16 | 2030 |
| MCR | Piccard | X-19 at BV 4, BVM | FS854 | Bin_7 | FS854-Bin_7 | 1625685 | 132 | 18714 | 39.3502 | 90.6475 | 5.7554 | Sulfurovum_17 | 1755 |
| MCR | Piccard | X-19 at BV 4, BVM | FS854 | Bin_9 | FS854-Bin_9 | 1826106 | 241 | 9032 | 38.6602 | 75.5396 | 4.3165 | Sulfurovum_18 | 2015 |
| MCR | Piccard | Shrimp Gulley 2, BSM | FS856 | Bin_37 | FS856-Bin_37 | 1934456 | 168 | 17281 | 36.2507 | 94.2446 | 4.3165 | Sulfurovum_19 | 2065 |
| MCR | Von Damm | near Main Orifice | FS866 | Bin_31 | FS866-Bin_31 | 1835174 | 321 | 6559 | 34.3603 | 74.8201 | 4.3165 | Sulfurovum_20 | 2095 |
| MCR | Von Damm | Old Man Tree | FS881 | Bin_43 | FS881-Bin_43 | 1943705 | 167 | 16326 | 38.8970 | 90.6475 | 2.8777 | Sulfurovum_21 | 2057 |
| MCR | Von Damm | Old Man Tree | FS881 | Bin_45 | FS881-Bin_45 | 1736799 | 161 | 14653 | 35.7433 | 79.8561 | 1.4388 | Sulfurovum_22 | 1915 |

**Supplementary Table 2.** All Sufurovum MAGs. Each Sulfurovum MAG recovered from the two hydrothermal sites is listed with its ID, number of contigs, length, GC content, completion, redundancy, and number of genes present.
